# Medicine

**Published:** 1895-09

**Authors:** R. Shingleton Smith


### Progress of the Medical Sciences.

### MEDICINE.

The recent meeting of the British Medical Association in London gives a convenient topic for our report, and, at the outset, it may be observed that the prevailing idea running through the various Presidential and general addresses, as well as the sectional discussions and papers, relates to the influence of micro-organisms and their products. This subject is necessarily the one which will chiefly occupy our attention. The President emphasised the fact that micro-biology and all that therein is, including the germ theory of disease, is not new, inasmuch as Harvey wrote on the scattering and propagation to a distance through the air of the seeds of epidemic, contagious, and pestilential diseases; and Sir Russell Reynolds further directed attention to the fact that microbes are not all pernicious in their action. He speaks of the beneficent microbe, and of the gradually-increasing appreciation of the fact that these lower forms of life exert, not necessarily mischievous, but, indeed, benignant influences on the human body, assisting normal functions, and even warding off the malign effects of other influences to which we are habitually exposed.[1]

Prof. Schäfer, in the address in Physiology, has placed before us a precise and lucid account of the action of products (not of bacterial origin but produced by chemical agencies in glands), and gives us the results of much good work which has recently been accomplished with regard to the production and neutralisation of toxins of internal origin.[2]

Sir William Broadbent, in the address in Medicine, after his excellent summary of the history of the growth of our art, and after having pointed out that the influence of both disease and therapeutics in the environment of the cell is a matter of chemistry, which must ultimately form the link between physiology, pathology, and therapeutics, further emphasises the fact that all vital actions are chemical actions, and that the key to therapeutics is to be found in the chemistry of vital processes; and then he rather hints that we may be carried away by fashion in medicine, and considers it to be inevitable that our imagination should be impressed by the revelation of the wonderful part which micro-organisms have been found to play in the genesis of disease.[3]

Dr. F. W. Pavy, the President of the section of Medicine, in his opening remarks, gave an outline of the history of

[1] *Brit. M. J.*, 1895, vol. ii., p. 263. [2] *Ibid.*, pp. 341, 373.
[3] *Ibid.*, p. 266.

microbes, toxins, and immunity. "It is," he said, "the toxin which leads to the generation of antitoxin, and toxin is produced by the bacillus, no matter whether it exists inside the system or is cultivated in a medium outside the body. Produced as the result of cultivation outside the body, it is susceptible of separation by filtration from the bacillus, and in this state the effect of its introduction into the system of one of the lower animals is to kill if used in sufficient quantity, and in smaller quantity to give rise to the production of antitoxin, which, with suitable arrangements, may be procured in a form to be susceptible of employment as a therapeutic agent."

The discussion on Diphtheria and its treatment by antitoxin was opened by Dr. Sidney Martin, who, after a preliminary statement on the two methods of treatment previously in vogue —the one by means of local applications which would loosen or remove the membrane from the throat, the other by remedies which would act upon the general symptoms of the disease—proceeded as follows: "Diphtheria may be defined as a membranous inflammation of a mucous membrane, usually of the respiratory passages, due to the invasion of the bacillus diphtheriæ, which does not enter the body but forms poisons in the membrane, which produce general symptoms, fever, &c., as well as a palsy due chiefly to a nerve degeneration." The presence of nerve degeneration is here for the first time insisted upon as *the* pathological test of the presence of this disease. Other membranous inflammations of the throat may be due to micro-organisms, but diphtheria is the only one which leads to nerve degeneration. The pathological test of the poisons found either in the bodies of patients dead of diphtheria or formed by the bacillus diphtheriæ outside the body is not that they produce death, but that they produce nerve degeneration. This degeneration is then the final and most accurate test of the presence and action of the bacillus diphtheriæ. This being the essential point in the pathology of the disease, it is the foundation of the recommendation of the new remedy, antitoxin—a recommendation based on scientific principles established first in the laboratory and afterwards at the bedside. It is the function of the antitoxin to counteract the chemical poisons found in diphtheria, which are the cause of the nerve degeneration. These poisons are of two classes: one found in the membrane, the other in the tissues; and these differ in many respects. "The pathological process in diphtheria must be considered in the following manner. When the membrane is formed, the bacilli growing in it, especially near the surface, secrete a ferment which, when absorbed into the body, produces by acting on the proteids of the body digestive products, the chief of which belong to the albumose class. It is not that the body is poisoned by a single large dose and then the action stopped

(although this may occur in certain cases), but it is that numerous small doses are, in the course of the disease, absorbed into the system, and are gradually producing their effects."[1] The chemical poison found in the membrane is of the nature of a ferment: no analysis is possible, but it has a profound effect in an infinitesimal dose, and it produces the symptoms and the pathological changes found in diphtheria. The poisons found in the tissues of patients dead of diphtheria differ from the poison in the membrane. They are of two kinds, the most important belonging to the class of digested proteids; viz., albumoses. The effects of injection of toxin, antitoxin, and albumoses were described and illustrated, and finally it was pointed out that after the antitoxin treatment of animals there was no fatty degeneration of the heart. The investigation, of which the details were described, tended to show that the antitoxin serum is capable of counteracting to a great extent the poisons which are found in the tissues of patients dead of diphtheria.

The evidence given by Prof. Von Ranke, of Munich, was very powerfully in favour of the new treatment. His statistics showed that the mortality had been reduced from 56.2 per cent. to 17.7. Various other details were given, and the remarkable fact announced that intubation was never found to be necessary if not required within the first twenty-four hours of treatment. Prof. Baginsky, of Berlin, gave a similar record, the mortality under serum treatment having been reduced from 41 to 15.6 per cent. Dr. Campbell Hall stated that he would not feel justified in treating a diphtheria patient in any other way than by the serum method as long as serum could be procured. Other speakers were unanimously in favour of the new treatment, and Dr. Sims Woodhead maintained that, in view of the number of experimental and clinical facts brought forward, a high degree of enthusiasm was thoroughly justified.

The report on antitoxin treatment in Germany,[2] obtained by collective investigation, on a total of 10,312 cases occurring in a period of six months from October 1st, 1894, gives very striking results. Of the whole number 5,833 were treated with serum, and 4,479 without it. The proportion of deaths in the former group was 9.6 in contrast to 14.7 per cent. in the latter. In 401 cases of children under the age of 2 years in which the serum treatment was used on the first and second day the mortality rate was 11.8, in contrast with 39.7 where under similar conditions it was not used. Of 2,556 children between 2 and 10 years of age, the death-rate was 4 per cent. after toxin treatment, instead of 15.2 per cent. in the other group. Over 10 years of age only 1 per cent. in a series of 696 cases died after early treatment, the mortality in the other group being 3.7 per cent. Other results, of which details have been given, are uniformly in favour of the serum treatment.

[1] *Brit. M. J.*, 1895, vol. ii., p. 462. [2] *Ibid.*, p. 495.

Dr. F. A. Dixey's statistics of diphtheria in London, from 1891 to 1895,[1] give further evidence in favour of the utility of modern treatment, inasmuch as he finds a diminution in mortality which has become apparent since the spring of 1894. The average weekly number of deaths from diphtheria for the whole year, which rose from 26.2 in 1891 to 36.2 in 1892, and to 62.8 in 1893, fell in 1894 to 51.4, and for the first half of the present year to 33.5. It is evident that from some quarter fatal diphtheria has received a very decided check, and this is not to be explained by any diminution in the occurrence of the disease, which has continued as prevalent as before.

The discussion of this subject, and the very striking results which are rapidly accumulating on all sides, make it incumbent on all who have to treat cases of diphtheria to profit by the experience already acquired. If ever clinical evidence is to be accepted as a basis for treatment we have it here in overwhelming abundance, and it will need much adverse criticism to weaken the evidence on which it becomes the bounden duty of the medical man to act in accordance with the results already acquired by scientific and clinical investigation. Dr. J. W. Washbourn, in the discussion on serum therapeutics, opened from the scientific standpoint by Dr. Klein,[2] makes the following state ment from a clinical point of view: "From a careful and critical examination of the statistics hitherto published, I have no hesitation in saying that they definitely prove the vast superiority of the antitoxin treatment over any other method of treatment that has hitherto been adopted."

The debate on acute lobar or croupous pneumonia, its etiology, pathology, and treatment, was opened by Dr. R. Douglas Powell, who gave an excellent *resumé* of the present aspects of the question. After expressing much regret that he could not record any practical abatement in the mortality of the disease, he emphasised the fact that probably 20 per cent. of the cases were attributable to surface-chills, contracted through hygienic imprudence, especially amongst those past middle life, and this imprudence was likely to be encouraged by the fatalistic and inadequate view that the reception of a microbe was alone sufficient to cause the disease. After having reviewed other conditions besides chills, such as depressed vitality, privation, the uræmic state, rheumatic conditions, diabetes, &c., Dr. Powell passed on to the question: how far was the reception and cultivation of a specific organism essential? Friedländer's, Fränkel's, and Klein's microbes were contending for the honour of disseminating pneumonia, the pneumococcus of Fränkel being most in favour. Undoubtedly pneumonia was a specific general (and therefore a microbic)

[1] *Brit. M. J.*, 1895, vol. ii., p. 531. [2] *Ibid.*, p. 406.

disease, but the pneumococcus could not as yet be altogether accepted as *the* germ of pneumonia. He pointed out that besides the prevalence of this coccus in pneumonia it had been also found in healthy saliva, in urine, in the exudation of meningitis, in ulcerative endocarditis, in otitis media, and conditions which have little resemblance to or association with pneumonia. In secondary inoculations septicæmia was the most common result, and this was too loosely accepted as valid evidence of specific virulence. Pneumonia might resemble such diseases as tuberculosis, erysipelas, acute rheumatic tonsillitis, and influenza, which, although possessing a specific organism, required for their efficient etiology certain additional conditions of impaired vitality, local and general, to enable the organism to become aggressive. After describing some varieties of the disease, and having reviewed the principal methods of treatment in vogue, Dr. Powell expressed himself in favour of the routine saline treatment as sufficient and useful for ordinary cases, whilst various drugs such as strychnine, caffeine, digitalis, and morphia were most valuable remedies for special indications. He does not encourage treatment of the pyrexia, and would consider a temperature up to 104° to be normal. The pyrexia of pneumonia is brief, normal to the disease, unfavourable to microbic activity, and inasmuch as the growth of this germ was checked by a temperature of 104°, it was not advisable to reduce it when not excessive, and then not below 102°. He was not able as yet to sanction the use of antitoxin in pneumonia, but he thought that it would probably be along these lines that progress would be made in the future.

Dr. Washbourn continued the discussion, and spoke on the specific character of the disease, its artificial production, and its variations in virulence, the modification of the type being masked by local action, as in meningitis. He believed that the sole cause of the symptoms was the formation and action of toxins, and that the crisis was due to the formation of a substance called an antitoxin. Dr. Auld, Dr. Dreschfeld, and Dr. Foxwell all spoke on the specific infectious character of the disease, and Dr. Tyson considered that the bacteriological examination of the subject served to explain the great differences clinically met with in various cases. Many other speakers commented on various details of treatment, and I took the opportunity of expressing regret that the meeting had not heard from Dr. Petresco, of Bucharest, some details of the means by which he is able to report a mortality of only two per cent. in a series of 1,300 cases, after treatment by large doses of digitalis, in contrast to the usual results of English hospital treatment which show a mortality of twenty to twenty-five per cent. Dr. Balfour, of Edinburgh, also spoke in favour of digitalis, in moderation, to rouse the failing heart, and stated that as much as 120 grains would often be required if continued till the crisis occurred. Professor Baümler, of Freiburg, eulogised the utility of cold bathing, but he did not approve of the use of medicinal antipyretics.

The third subject for debate in the Medical Section was opened by Dr. Cheadle—the causes of acute rheumatism, and its relation to other affections. At first sight it does not appear to have much connection with bacteria and their products, but the theoretical existence of a poison as a cause of rheumatism is a matter of ancient history, although it still remains to be proved what the nature and origin of that poison may be. It was inevitable that modern investigations on microbes and toxins should re-open the question as to the nature of the rheumatic poison, and the debate introduced so ably by Dr. Cheadle will have done something towards a solution of these questions.

The first point emphasised was that acute rheumatism is a general disease, of which arthritis is but one of many symptoms. The cardiac affection of chorea was probably rheumatic, it being organic, and endocarditis being the most constant change in fatal chorea. Pericarditis and endocarditis were almost exclusively rheumatic, and so were the exudative erythemata. After a review of the influences of heredity and season, with especial emphasis on chill as a factor, a notice of the exhaustive researches of Dr. Newsholme associating the disease with low subsoil water and an unusually high soil temperature, led up to the idea that some general systemic influence existed, and that this must be a toxæmia rather than a neurosis. The toxæmic agent produced some more or less localised irritant effect on one or other of the tissues; it might be a chemical poison, it might be a product of a micro-organism. It was not uric acid (Haig), nor was it lactic acid (Prout), or a combination of these (Latham). The arguments in favour of invasion by a micro-organism had been forcibly given by Dr. Newsholme in his Milroy Lectures. Contagion must be rare; the disease had not been produced by inoculation in man, but nevertheless all the varied phenomena of the disease were suggestive of an infectious origin, although the specific organism had not yet been discovered.[1]

Sir Dyce Duckworth accepted the infective theory to a limited extent, and was looking for a more exact proof of the truth of the theory, but he considered that a diathetic disposition is also a necessary factor, and that this was transmitted by heredity; the infective theory failed as yet to account for all the features of the malady. Dr. Archibald Garrod considered that there was no evidence whatever in favour of the lactic acid theory, and that there could be little doubt as to the infective nature of the disease. The long-continued course, and the way in which the disease crops up like malaria, were more analogous to syphilis than to

[1] *Lancet*, 1895, vol. ii., p. 435.

an acute infectious disease. He suggested that the temperature curve of rheumatism was the result of the local changes, and that the chorea might be the result of a secondary toxin of microbic origin by a process resembling that of post-diphtheria paralysis. Dr. A. Mantle referred to his views on this subject published nine years ago, and gave instances of the epidemic prevalence of rheumatism, also of its frequent association with epidemic scarlet fever, and with infective sore throat. He had found cocci and bacilli in the joints and blood-serum of patients with acute rheumatism. Dr. Newsholme wished to abolish the term acute rheumatism, and maintained that clinical, pathological, and therapeutic considerations were strongly in favour of the view that rheumatic fever was a specific febrile disease. The evidence in many countries was in favour of an intermittent and epidemic prevalence, and in Norway there appeared to have been a gradual spread from a given centre. Dr. Haig again expressed his well-known views on uric acid, and believed that in all doubtful points the uric acid theory gives a satisfactory solution to the problems. Dr. S. Mackenzie observed that in several other microbic diseases no microbe had yet been found, and that chorea is just as much rheumatism as is acute polyarthritis. Dr. D. B. Lees observed that the occasional loss of knee-jerk in chorea, just as in diphtheria, was likely to result from the action of a blood poison. He suggested the analogy of diphtheria and of tetanus, in which a local multiplication of micro-organisms occurred and a general poisoning of the system by a toxin. Various other speakers were for the most part in favour of the microbic theory as an important factor in the etiology of this disease.[1]

R. Shingleton Smith.

[1] *Lancet*, 1895, vol. ii., p. 435.

